# Improving Function in Cavernous Sinus Meningiomas: A Modern Treatment Algorithm

**DOI:** 10.3389/fneur.2020.00652

**Published:** 2020-07-24

**Authors:** André Beer-Furlan, Blake H. Priddy, Ali O. Jamshidi, Ammar Shaikhouni, Luciano M. Prevedello, Leo Ditzel Filho, Bradley A. Otto, Ricardo L. Carrau, Daniel M. Prevedello

**Affiliations:** ^1^Department of Neurological Surgery, Rush University Medical Center, Chicago, IL, United States; ^2^Department of Neurological Surgery, Wexner Medical Center, The Ohio State University College of Medicine, Columbus, OH, United States; ^3^Department of Neurological Surgery, Indiana University, Indianapolis, IN, United States; ^4^Department of Radiology, Wexner Medical Center, The Ohio State University College of Medicine, Columbus, OH, United States; ^5^Department of Otolaryngology—Head and Neck Surgery, Wexner Medical Center, The Ohio State University College of Medicine, Columbus, OH, United States

**Keywords:** cavernous sinus, meningioma, endoscopic endonasal, skull base, parasellar

## Abstract

**Background:** The efficacy and safety of radiosurgery led to paradigm shift in the management of cavernous sinus meningiomas. Nevertheless, patients are still significantly affected by cranial nerve deficits related to the mass effect of these tumors. Our management strategy involves the combination of a functional surgical decompression followed by radiation therapy.

**Methods:** We reviewed a single institution's cohort of patients who underwent endoscopic endonasal decompression (EED) for symptomatic meningiomas primarily involving the cavernous sinus (CS) from 2010 to 2016. The preoperative neuro-ophthalmological exam was compared to the 1- and 6-month postoperative exams. The patient's length of hospital stay, complications, and radiological and clinical follow-up were noted.

**Results:** A total of 17 patients underwent EED for CS meningiomas that fit our radiological criteria. The final outcome at the 6-month visit showed five patients (62.5%) with normalization of deficit and three patients (37.5%) with partial improvement of the CNII deficit. Out of the 12 patients who had cavernous sinus cranial nerves (CSCN) deficits, the final outcome at the 6-month visit showed four patients (33.33%) with normalization of deficit, seven patients (58.3%) with partial improvement, and one patient (8.33%) with no improvement. There were no intraoperative complications.

**Conclusion:** The EED for CS meningiomas is a valuable technique when addressing acute/subacute CNII and CSCN deficits. This conservative surgical approach showed good functional outcomes, low morbidity, and low complication rates. However, it does not exempt the need for radiosurgery/radiation therapy for control of tumor growth.

## Introduction

The management of cavernous sinus (CS) meningiomas changed significantly in the past two decades. The growing knowledge of skull base anatomy and microsurgical approaches led to attempts of aggressive resection of these tumors in the 1980 and 1990's. Suboptimal reports on postoperative cavernous sinus cranial nerves (CSCN) function resulted in a gradual reconsideration of the management strategy (1, 2). Concomitantly, stereotactic radiosurgery and stereotactic fractionated radiotherapy became an attractive alternative as adjuvant or primary treatment, with many series showing control rates exceeding 90% over 5 years (3, 4).

The paradigm shift in the management of CS meningiomas was driven by the efficacy and safety of radiosurgery, histological evidence of cranial nerve (CN) infiltration by meningiomas, and greater consideration of the impact of postoperative outcomes on the patients' quality of life (2, 5–7).

Different conservative surgical approaches with the goal of safe tumor debulking or simply CS decompression reported promising results in recovery and preservation of CSCN function (8–11). Hence, the combination of a functional surgical decompression with adjuvant radiation therapy for tumor growth control appears to be an optimal management strategy for CS meningiomas (5, 7).

In this context, we describe and discuss our treatment algorithm for symptomatic CS meningiomas with a focus on the endoscopic endonasal decompression (EED) technique and outcomes.

## Methods

In this retrospective study, we reviewed a single institution's cohort of patients who underwent EED for symptomatic meningiomas involving the CS from 2010 to 2016. The study was performed under Institutional Review Board approval and did not require patient consent. The information collected from patient's electronic medical records included demographics, presenting symptoms, neuroophthalmological exam, operative notes, postoperative course, histopathological diagnosis, laboratory data, clinical follow-up, and radiological imaging.

Patients with meningiomas located primarily in the CS were selected. Based on magnetic resonance imaging (MRI) analysis, we only included patients that had at least 50% of the total tumor volume within the CS and without significant cisternal tumor extension that could explain the patient's CN deficit. Meningiomas of other locations that were secondarily invading the CS were not included.

### Imaging Analysis

The imaging and volumetric assessment and analysis were independently performed by an experienced neuroradiologist with access to all imaging sequences.

**Figure 1:**
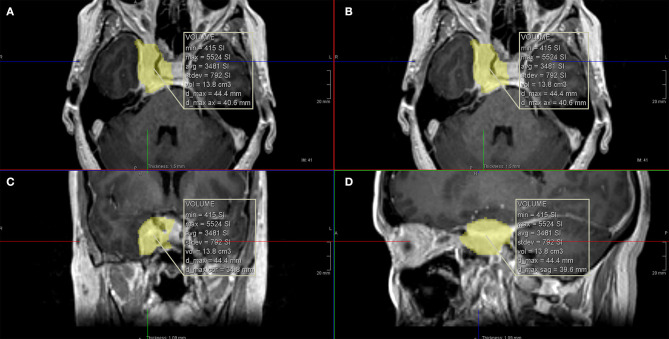
Calculation of the total tumor volume using computer software. **(A,B)** Axial T1 with gadolinium images; **(C)** coronal T1 with gadolinium image; **(D)** sagittal T1 with gadolinium image.

**Figure 2:**
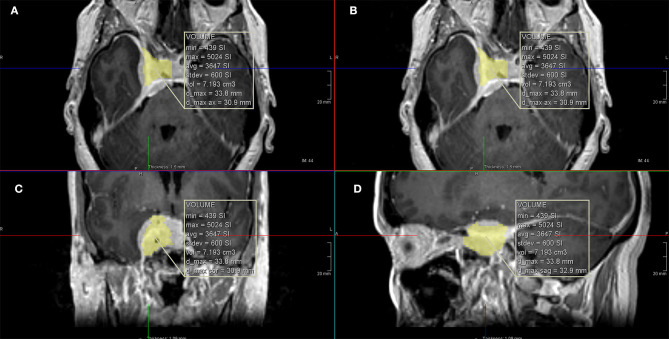
Calculation of the intracavernous volume of tumor using computer software. **(A,B)** Axial T1 with gadolinium images; **(C)** coronal T1 with gadolinium image; **(D)** sagittal T1 with gadolinium image.

For the volumetric assessment, submillimetric postcontrast images were used and processed on IMPAX Volume Viewing 3.0 (AGFA Healthcare, Mortsel-Belgium). Extracranial extension of the meningioma (intraorbital, infratemporal fossa, nasopharynx, or carotid space) were evaluated separately. All components of the tumor were measured manually. If the tumor crossed the midline, only the symptomatic side was measured considering the medial border as a virtual sagittal line at the level of the septum pellucidum and pituitary infundibulum. The medial border of the cavernous component of the tumor was considered a virtual sagittal line along the medial border of the cavernous internal carotid artery (ICA). The lateral border of the cavernous component was considered the lateral dural reflection of the CS that was determined by the differential contrast enhancement and low T2 signal in this region (Figures 1, 2).

### Management Strategy

**Figure 3:**
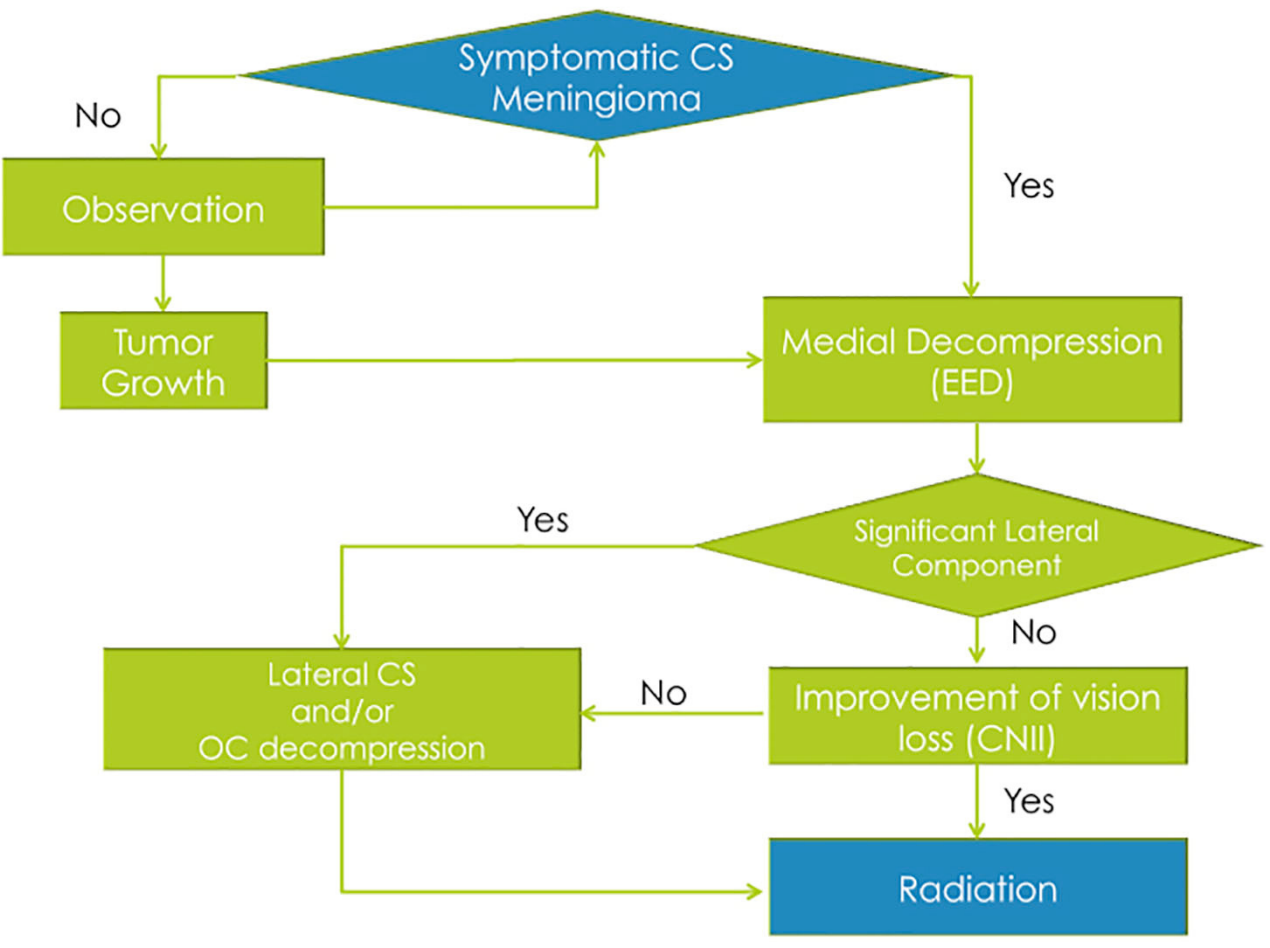
Summary of the “functional skull base surgery” strategy used in cavernous sinus meningiomas.

Our management strategy for CS meningiomas involves prioritizing functional preservation of cranial nerves and control of tumor growth rather than tumor resection (Figure 3). Asymptomatic patients are observed and periodically scanned for assessment of tumor growth. A combination of surgery for CS decompression followed by stereotactic radiosurgery or radiation therapy is offered to all symptomatic patients. The EED of the bone overlying the sella, CS, and optic canal (OC) are performed first. If feasible, tumor biopsy is done for pathological diagnosis.

If there is no significant lateral extra-CS component of the tumor, the patient is referred to the radiation oncologist.

If there is a significant component of the tumor laterally, outside of the CS, then a craniotomy for lateral CS decompression and tumor resection is planned within 2 months from the EED. In cases of vision loss with no improvement after EED of the OC, the open frontolateral approach also includes complete optic nerve decompression. After the complete surgical management, the patient is sent to adjuvant radiation therapy for the residual CS tumor.

### Operative Technique

The endoscopic endonasal approach to the sellar and parasellar regions have been extensively described in the literature (12–16). In brief, the exposure is initiated with a middle turbinectomy, bilateral mucosal rescue septal flaps (17), posterior septectomy, wide bilateral sphenoidotomies, and ipsilateral posterior ethmoidectomies.

A high-speed drill is used to address meningioma-associated hyperostosis of the bone overlying the medial CS. The bone overlying the sellar floor and medial CS all the way anterior to the superior orbital fissure is drilled until it is very thin and mobile. Cottle dissector and microdissectors are used to safely separate the bone from dura mater and remove it. When the patient is symptomatic from OC invasion of the meningioma, we proceed with an extensive (at least 180°) medial decompression of the OC with similar drilling technique.

**Figure 4:**
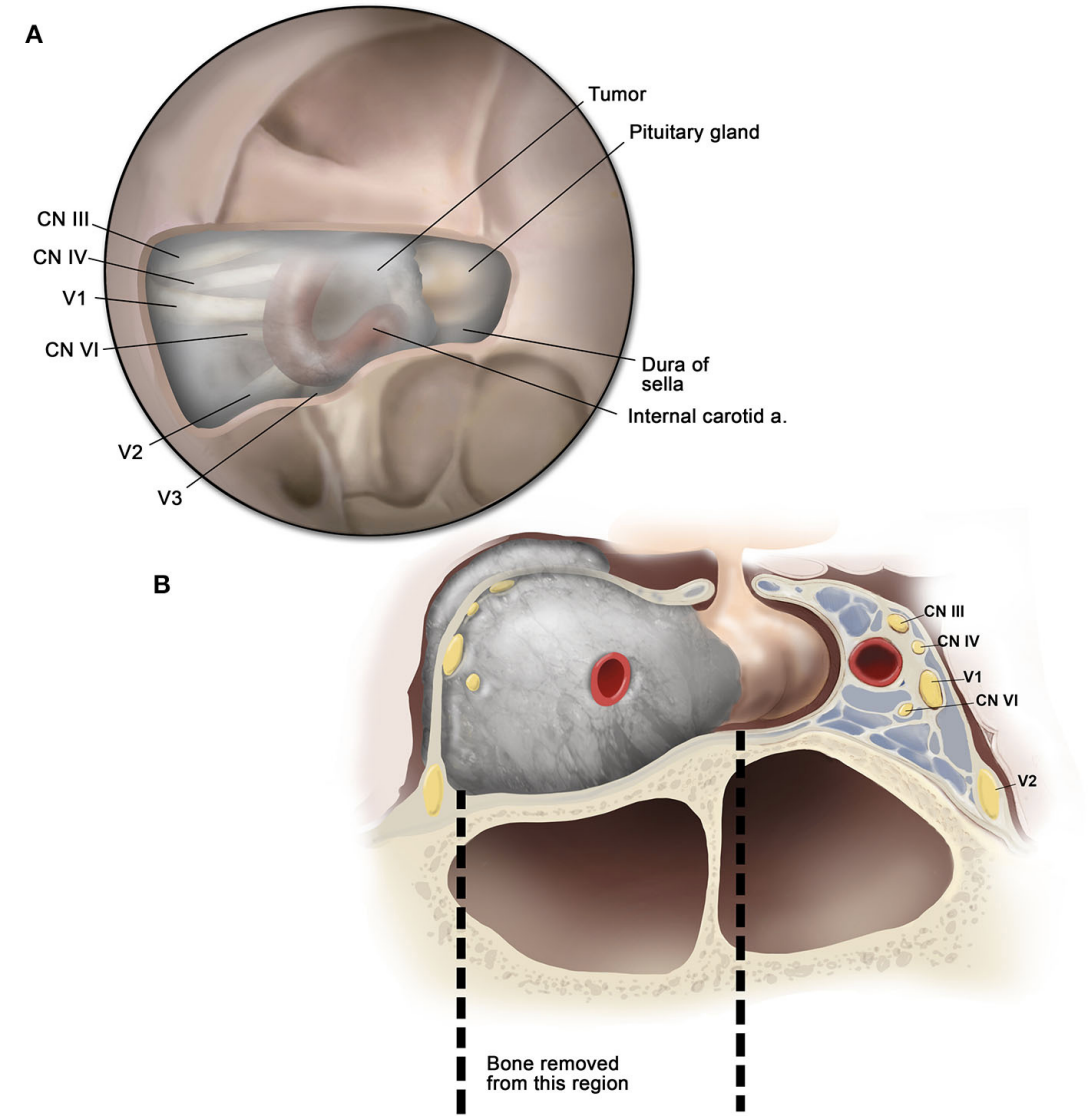
Illustration of the surgical anatomy. **(A)** Endoscopic view showing the cavernous sinus anatomy after removal of the bone overlying the sella and cavernous sinus; **(B)** coronal cross-section illustration demonstrating the area of bone decompression.

**Figure 5:**
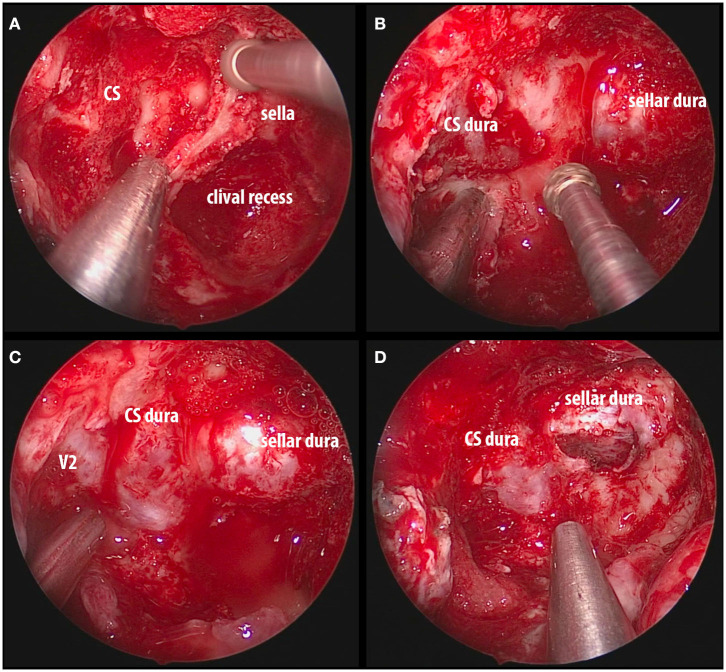
Intraoperative images of endoscopic endonasal decompression for a right side CS meningioma. **(A)** Drilling down the intersphenoid septum and accessory septa flush with the ventral skull base; **(B)** exposure of the sellar dura and CS dura with a bridge of bone overlying the cavernous internal carotid artery; **(C)** complete decompression of the bone overlying the sella, medial SOF, and CS; **(D)** opening the sellar interdural space (between the periosteal and meningeal layers of dura) that is occupied by the tumor and it is considered one of the safe zones for biopsy. CS, cavernous sinus; SOF, superior orbital fissure; V2, maxillary nerve.

**Figure 6:**
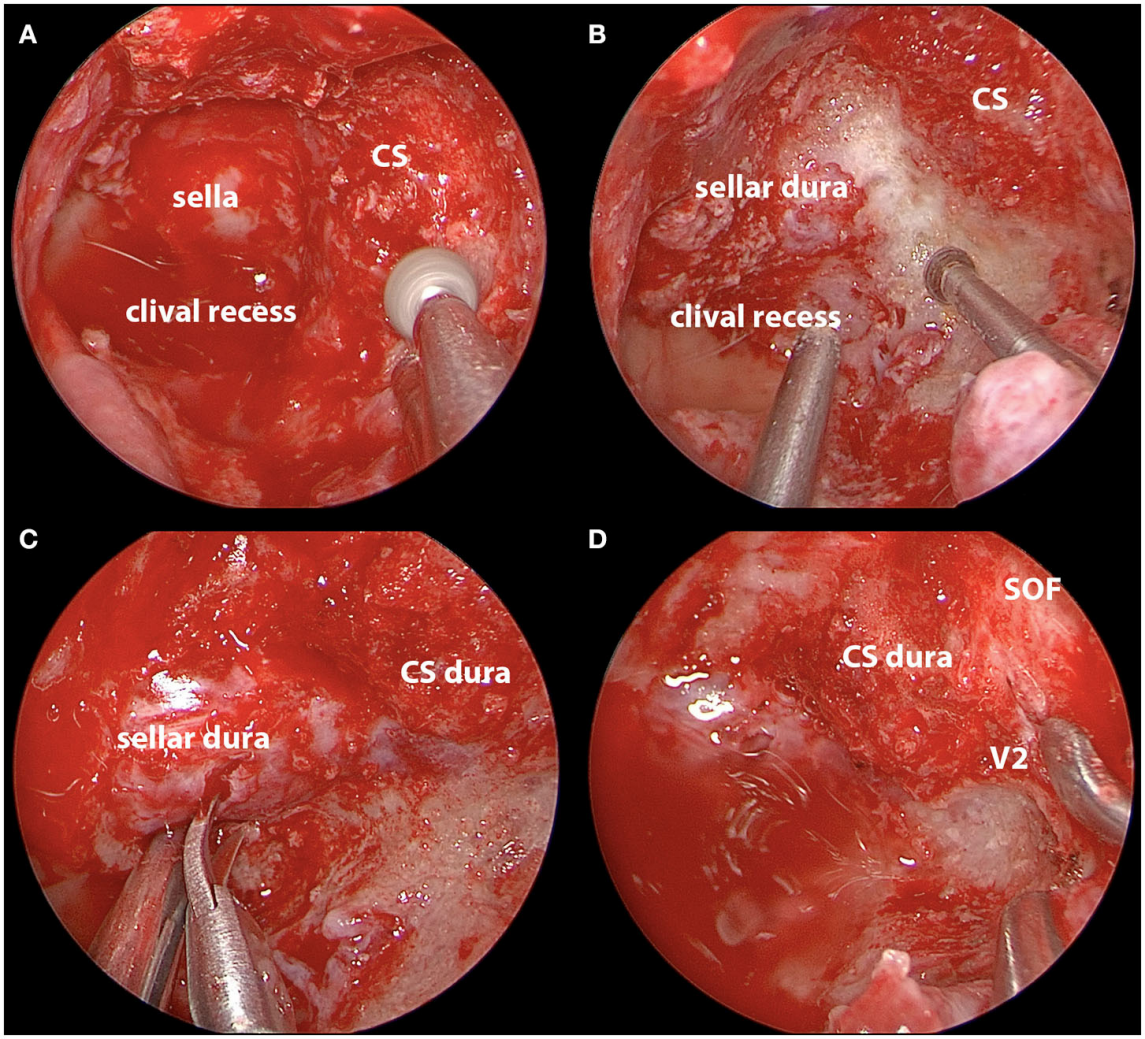
Intraoperative images of endoscopic endonasal decompression for a left side CS meningioma. **(A)** Drilling down the hyperostotic bone overlying the CS; **(B)** exposure of the sellar dura; **(C)** opening the sellar interdural space after complete decompression; **(D)** decompression of bone overlying the sella, medial SOF, and CS. CS, cavernous sinus; SOF, superior orbital fissure; V2, maxillary nerve.

**Figure 7:**
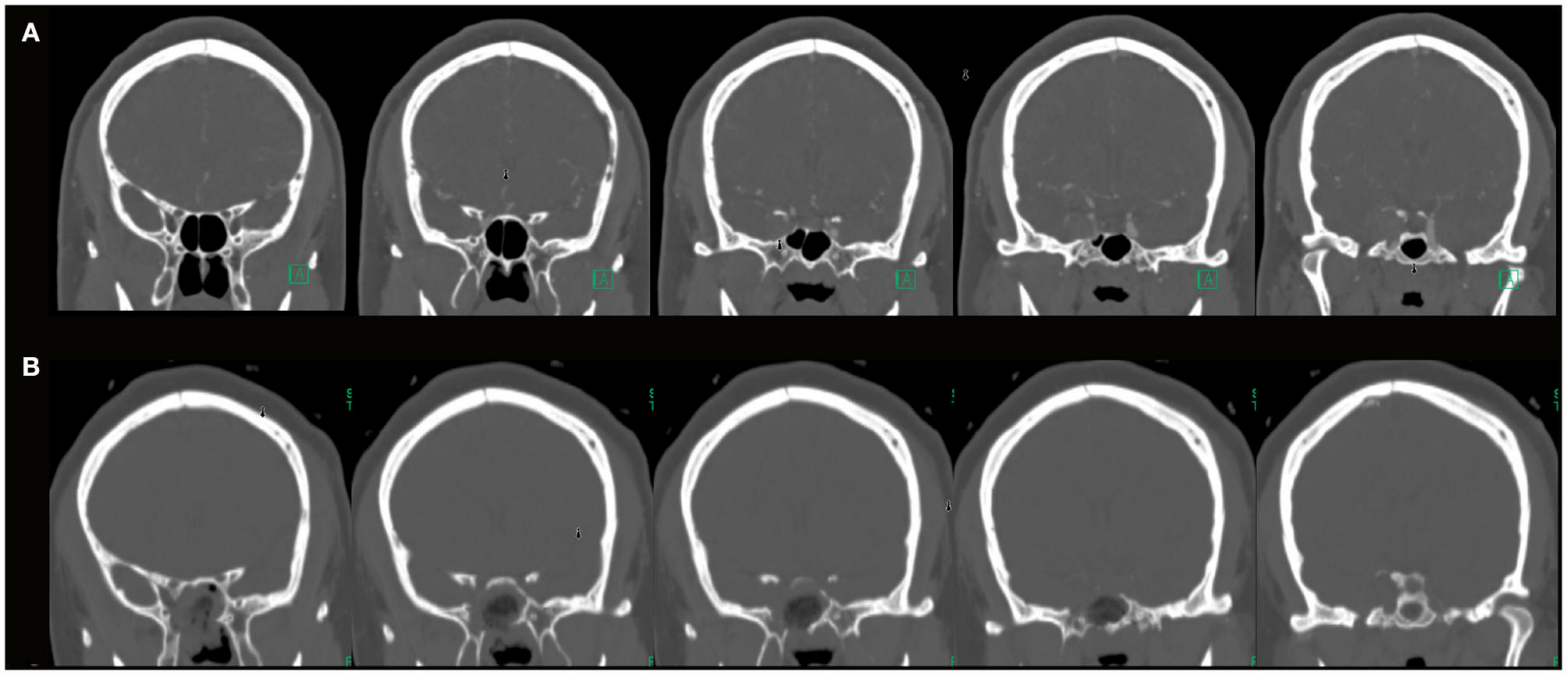
**(A)** Preoperative coronal CTA images (bone window) of a patient with a right side cavernous sinus meningioma; **(B)** postoperative coronal CT images (bone window) showing the area of bone decompression on the sella, medial aspect of the superior orbital fissure and cavernous sinus.

The procedure is essentially extradural with removal of the entire medial wall of the superior orbital fissure and CS (Figure 4). The dura is then opened in the two safe areas (at the level of V2 and sella) for tumor biopsy and further decompression. Usually, the CS meningiomas grow from one CS to the other by projecting in between the two layers of the dura. Thus, one can identify, biopsy, and debulk the tumor at the sellar level without affecting the pituitary gland (Figures 5, 6). Tumor resection and debulking is very conservative since the main goal of the procedure is to achieve bone decompression and pathology diagnosis. It usually will not exceed more than 10% of tumor volume since the preservation of function is a priority of this surgery (Figure 7).

As there is rarely cerebrospinal fluid (CSF) spaces exposure in these cases, the exposed dura is covered with muco-periosteal graft from the middle turbinate resected at the beginning of the procedure or a nasal septal flap. Posterior nasal packing is placed to buttress the skull base reconstruction.

### Outcomes

The patient's length of hospital stay after the EED procedure and any complications during the postoperative course were noted. Neuro-ophthalmological assessment included visual acuity, perimetry, and ocular movement, with or without diplopia at maximal gaze in all directions. The visual deficits were differentiated into optic nerve (CNII) and CSCN (CNIII, oculomotor; CNIV, trochlear; and CNVI, abducens) deficits. CNII deficits were related to loss of visual acuity and visual field defects. CSCN deficits were related to ocular movement issues and diplopia. The preoperative neuro-ophthalmological exam was compared to the 1- and 6-month postoperative exams.

The modified grading system for binocular vision (ocular motility) was used to compare outcomes for the patients with CSCN deficits (8).

The management after the EED was recorded including the need and timing for a craniotomy for lateral CS decompression, stereotactic radiosurgery/radiation therapy and timing after EED, tumor growth during follow-up, and neurological status on last follow-up visit.

## Results

### Clinical Features

A total of 17 patients underwent EED for CS meningiomas that fit our radiological criteria. Three were male (17.64%), and 14 were female (82.36%). The age range for the patients was 29–84 years, with an average of 52.9 and median of 52 years.

The most common presenting symptoms were double vision/diplopia in 12 patients (70.6%), visual loss in 8 patients (47.1%), ocular and periorbital pain in 7 patients (41.2%), and facial numbness/paresthesias in 3 patients (17.6%).

In regard to the preoperative CN deficits, five patients (29.41%) had only an optic nerve (CNII) deficit, nine patients (52.94%) had only one or more deficit of a CSCN, and three patients (17.65%) had CNII and a CSCN deficit.

The time of onset of symptoms at presentation varied from 1 to 124 months (average of 14.3 months and median of 4.5 months).

The range for whole tumor volume was 2.89–13.8 cm^3^ (average of 6.66 cm^3^ and median of 5.73 cm^3^). The intra-CS volume component of the tumor ranged from 1.95 to 7.42 cm^3^ (average of 4.34 cm^3^ and median of 3.89 cm^3^). The percentage of the intra-CS component of the tumor ranged from 51.65 to 75.29% (average of 65.89% and median of 67.16%).

None of the patients had received stereotactic radiosurgery/radiation therapy before surgery, and the EED was the first treatment modality in all patients.

There were no intraoperative complications. The pathology revealed that 12 patients (70.59%) had a meningioma WHO grade I. In four patients (23.53%), pathology was reported only as “meningioma.” There was one patient (5.88%) that tumor biopsy was not obtained. The Ki67 index was <1% in three patients, 2% in four patients, 3% in one patient, 4% in one patient, 5% in one patient, and not available in seven patients.

### Outcomes of Cranial Nerve Deficits

All of the eight patients that had CNII deficits experienced some degree of improvement of vision at the 1-month follow-up. At 6-month follow-up, one patient had stable vision exam and seven patients had further improvement. The final outcome at the 6-month visit showed five patients (62.5%) with normalization of deficit and three patients (37.5%) with partial improvement of the CNII deficit.

Out of the 12 patients that had CSCN deficits, 11 experienced some degree of improvement at 1-month follow-up. The same 11 patients experienced further improvement at the 6-month follow-up. The final outcome for CSCN deficit at the 6-month visit showed four patients (33.33%) with normalization of deficit, seven patients (58.3%) with partial improvement, and one patient (8.33%) with no improvement. The patient who did not have any improvement was the same patient who presented with more than 10 years (124 months) history of CSCN deficit (Table 1).

**Table 1:** Detailed cranial nerve outcomes of the series with the timeline of endoscopic endonasal decompression (EED), craniotomy, and radiation therapy.

	**Age**	**Gender**	**Presenting symptoms**	**CN II at 1 month after EED**	**CN II at 6 month after EED**	**CSCN at 1 month after EED**	**CSCN at 6 months after EED**	**Craniotomy after EED**	**Interval between EED and craniotomy (months)**	**Adjuvant radiation therapy**	**Radiation after achieving best CN II/CSCN outcome**
Patient 1	33	M	Double vision and ocular/periorbital pain.	ND	ND	Improvement	Further improvement (partial)	No	N/A	Yes	Yes
Patient 2	40	F	Double vision and facial paresthesia	ND	ND	Improvement	Further improvement (partial)	Yes	18	Yes	Yes
Patient 3	44	F	Double vision.	ND	ND	Improvement	Normalization	No	N/A	Yes	Yes
Patient 4	60	F	Double vision and facial paresthesia.	ND	ND	No improvement	No improvement	No	N/A	Yes	No
Patient 5	61	F	Visual loss, ocular/periorbital pain and facial paresthesia.	Improvement	Further improvement (partial)	ND	ND	Yes	2	No	N/A
Patient 6	52	F	Visual loss.	Improvement	Normalization	ND	ND	Yes	1	Yes	No
Patient 7	84	F	Visual loss, double vision, and ocular/periorbital pain	Improvement	Further improvement (partial)	Improvement	Further improvement (partial)	No	N/A	Yes	Yes
Patient 8	65	F	Visual loss.	Improvement	Stable	ND	ND	No	N/A	No	N/A
Patient 9	50	M	Visual loss, double vision, and ocular/periorbital pain	Improvement	Normalization	Improvement	Improvement (partial)	No	N/A	No	N/A
Patient 10	36	F	Double vision.	ND	ND	No improvement	Improvement (partial)	Yes	5	Yes	Yes
Patient 11	38	F	Double vision.	ND	ND	Improvement	Normalization	No	N/A	Yes	Yes
Patient 12	57	F	Double vision.	ND	ND	Improvement	Normalization	No	N/A	Yes	Yes
Patient 13	29	F	Double vision and ocular/periorbital pain.	ND	ND	Improvement	Normalization	Yes	2	Yes	No
Patient 14	53	F	Visual loss and ocular/periorbital pain.	Improvement	Further improvement (partial)	ND	ND	Yes	5	Yes	Yes
Patient 15	74	F	Visual loss.	Improvement	Normalization	ND	ND	No	N/A	No	N/A
Patient 16	51	F	Visual loss, double vision, and ocular/periorbital pain	Improvement	Normalization	Improvement	Further improvement (partial)	Yes	2	Yes	Yes
Patient 17	73	M	Double vision.	ND	ND	Improvement	Further improvement (partial)	Yes	2	No	N/A

*ND, no deficit*.

When the ocular motility grading system was applied to the 12 patients with CSCN deficit who underwent EED, we found that preoperatively two patients had “Good” function, six patients had “Fair” function, and four patients had “Poor” function. At the 6-month postoperative follow-up, we found that four patients had “Excellent” function, seven patients had “Good” function, and one patient had “Poor” function (Tables 2, 3).

**Table 2:** Comparison between preoperative and postoperative cavernous sinus cranial nerves (CSCN) outcomes based on the ocular motility grading system.

**Postoperative function**	**Preoperative function**			
	**Excellent (*n* = 0)**	**Good (*n* = 2)**	**Fair (*n* = 6)**	**Poor (*n* = 4)**
Excellent (*n* = 4)	0	2	2	0
Good (*n* = 7)	0	0	4	3
Fair (*n* = 0)	0	0	0	0
Poor (*n* = 1)	0	0	0	1

*The deficits were graded into: “Excellent,” no diplopia, no ptosis; “Good,” no diplopia in primary gaze, but diplopia out of primary gaze positions, mild ptosis; “Fair,” diplopia in primary gaze; “Poor,” moderate to severe ptosis, nearly total ophthalmoparesis*.

**Table 3:**
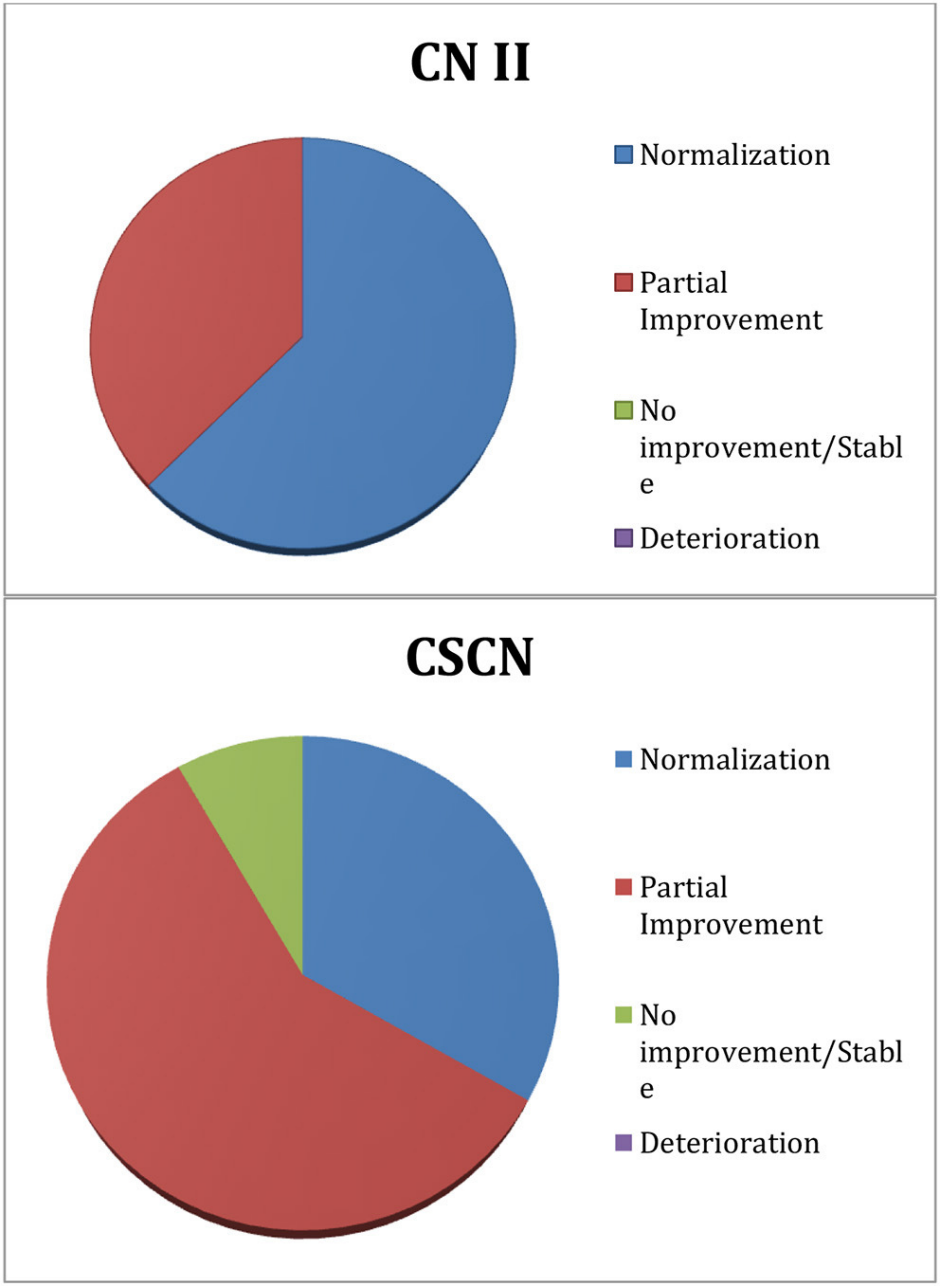
Visual (CNII) and extraocular movement (CSCN) outcomes at 6 months.

### Postoperative Course and Follow-Up

The length of hospital stay varied from 1 to 8 days (median of 2 days). There was one postoperative complication (5.88%) directly related to the surgery. This patient had a low flow cerebrospinal fluid leak that also resulted on his prolonged hospital stay of 8 days. The patient was successfully treated with a lumbar drain. There was one patient who developed an indirect complication, a postoperative hypoxic respiratory distress, which did not affect his recovery or timing of hospital discharge.

Additional craniotomy for decompression of the optic nerve and/or the lateral extra-CS component of the tumor was performed in eight patients (47.06%). The interval between the EED and the craniotomy ranged from 1 to 18 months (average of 4.6 months and median of 2 months). Of note, the heterogeneity in timing of the craniotomy and/or radiation therapy following EED is solely due to patient compliance to additional treatment. Some patients refused additional treatment due to symptoms improvement after EED.

Adjuvant radiation therapy was delivered in the form of stereotactic fractionated radiotherapy or radiosurgery. Out of the 17 patients, 12 (70.58%) received adjuvant radiation therapy. There were only three patients in which postoperative radiation therapy was started before the patient achieve their best cranial nerve (CNII and/or CSCN) outcome.

More than 6-month follow-up records were available in 15 patients. It ranged from 7 to 78 months (average of 33.8 months and median of 33 months). Radiological follow-up revealed tumor growth after surgical treatment in three cases that did not receive adjuvant radiation therapy right after surgical treatment. Two of these patients refused adjuvant radiation therapy. The third patient initially opted not to received radiation therapy but ended up receiving it in a delayed fashion (2 years after surgical treatment) only after evidence of tumor growth.

The long-term cranial nerve deficit (CNII and/or CSCN) outcome demonstrated clinical worsening in 3 out of the 15 patients who were followed up after the 6-month mark. One out of these three patients did not receive adjuvant radiation therapy.

### EED Subgroup

Nine patients were part of a subgroup who underwent EED as the only surgical treatment (not requiring additional craniotomy). In regard to the preoperative CN deficits of this subgroup, two patients had only CNII deficit, five patients had only one or more deficit of a CSCN, and two patients had CNII and a CSCN deficit.

The final CNII outcome at the 6-month visit of the “EED only” subgroup showed two patients (50%) with normalization of deficit and two patients (50%) with partial improvement of the CNII deficit. The final outcome for CSCN deficit at the 6-month visit of the “EED only” subgroup showed three patients (42.86%) with normalization of deficit, three patients (42.86%) with partial improvement, and one patient with no improvement that presented as an outlier (124 months after symptoms onset).

Of note, the three patients with complete resolution of CSCN deficit achieved the best outcome before radiation therapy. The two patients with complete resolution of CNII deficit refused radiation therapy due to improvement in symptoms.

## Discussion

The natural history of CS meningiomas is difficult to ascertain. It is believed that CS tumors represent 1% of all intracranial tumors, 41% of which are meningiomas (5, 18). Despite being relatively uncommon, CS meningiomas represent a subset of intracranial tumors that continue to pose a formidable challenge to neurosurgeons.

The idea of combining conservative surgery and radiation therapy for the management of these tumors is not novel, and it has shown promising outcomes. In 2006, Couldwell et al. (9) reported the strategy of conservative surgery followed by radiosurgery or fractionated radiotherapy in 11 patients with CS meningiomas. They performed a frontotemporal craniotomy for selective extra- and inter-CS tumor removal to decompress the CNs and reduce the overall tumor volume that would be treated with postoperative radiation. Although the study had a small sample size, three of five patients with eye motility difficulty and two of four patients with visual loss improved, and no patient suffered a new CN deficit after surgery.

Akutsu et al. (8) reported in 2009 their results using a conservative microscopic transsphenoidal surgical decompression in 21 patients with CS meningiomas. The sellar and part of the CS bone were removed, the dura was opened, and modest tumor debulking was performed. Overall, 32 of 34 CN deficits improved, and there was no worsening. Tumor control was 100% at median follow-up of 65 months.

Lobo et al. (10) published in 2015 a series of 15 patients in whom the EED was used to treat meningiomas involving the parasellar region (sella, CS, Meckel's cave, and clivus). In their series, 31 preoperative CN palsies related to the tumor were documented in 12 patients (80%). After the surgery, 13 CN palsies (42%) improved or resolved, and 18 CN palsies (58%) remained stable. One patient developed a new permanent abducens nerve palsy directly related to the surgery. No other patients had worsening of an existing cranial neuropathy secondary to surgery.

A more recent publication of the same group (11) demonstrated the results of endonasal bony decompression and partial tumor removal in 20 patients with parasellar and petroclival meningiomas. The authors divided their patient population in subgroups based on prior treatment. There were 14 patients without and 6 patients with previous debulking and radiation therapy. The median follow-up was 57 months.

Of the six who undergone optic canal bony decompression, vision improved in the postoperative period in four patients (66%) and had remained stable in two (33%), one of whom had undergone preoperative radiosurgery. Eight patients, who had presented with complete or partial ophthalmoplegia secondary to meningioma invasion of the CS, had undergone bony decompression of the affected CS with selective medial CS tumor debulking. At the latest follow-up examination, the ophthalmoplegia had improved in two patients (25%) and had remained stable in three (38%). However, ocular motility had worsened in three patients (38%), two of whom had received preoperative SRS and one who had developed a new and permanent CN VI palsy immediately after surgery. Their ophthalmologic results were worse than what we found in our study; however, their series was less selective in terms of tumor size, location, and prior treatment.

Meningiomas involving the CS are complex entities and may have heterogenous characteristics. We sought to include only tumors primarily located within CS and without significant cisternal extension that could explain the patient's CN deficit. By having selective inclusion criteria, we believed that the CNII and CSCN function outcomes would be a true representation of the EED effects.

Our management algorithm for CS meningiomas combines the different strategies previously published (8, 9). It is given preference to the EED due to the anatomical advantage and low morbidity of the transsphenoidal route to access the ventral–medial CS. If needed, a frontotemporal craniotomy is performed at a second stage to address the extra/inter-CS lateral component of the tumor and completes a 270° decompression around the optic canal. In our series, a subsequent craniotomy was required in 47.06% of the patients. The goal was to ensure maximal CS and optic canal decompression achieved with minimal CN manipulation before delivering stereotactic radiosurgery or radiotherapy.

The use of the endoscope and experience with expanded endoscopic endonasal skull base surgeries permitted us to do a more aggressive bone resection than what was described on the article by Akutsu et al. (8). Our technique involves bone removal of the sella and CS all the way to the superior orbital fissure and maxillary strut. In cases of CNII involvement, our medial OC decompression is also extensive from the medial falciform ligament to the orbital apex.

### Surgical Outcomes

The majority of our patients were women, and CSCN/CNII deficits were the main presenting symptoms. The median time of symptoms onset at presentation was 4.5 months, with one patient being an outlier and presenting after 124 months.

The EED demonstrated to be a safe procedure with no intraoperative complications. It also showed low morbidity with only one case of postoperative low flow CSF leak. Pathology diagnosis of meningioma was achieved in 94.12% of the patients. Postoperative management was similar to advanced sinus surgery patients. The majority of the cases were discharged home on postoperative day 1 or 2.

CNII deficit outcomes showed that all patients experienced improvement as early as 1 month after surgery. At the 6-month follow-up, 100% of the eight patients with preoperative CNII symptoms had some improvement (five patients with normalization and three patients with partial improvement).

The results also showed that 91.63% of the 12 patients with CSCN deficits experienced improvement at 1-month mark. At 6-month follow-up, the improvement rate remained the same with four patients (33.33%) with normalization (“Excellent” grade) and seven patients (58.3%) with partial improvement to at least a “Good” grade.

The only patient who maintained his “Poor” grading postoperatively presented to us after 124 months of symptoms. Although discussed with the patient the low chance of improvement of his CN deficits given the late presentation, we still offered conservative surgical treatment based on the good results obtained with previous patients.

Our outcomes for CNII/CSCN function are better than previous series published in the literature (8, 10). We believe that this is a result of this article's radiological inclusion criteria and aggressive medial bone decompression of the CS and/or OC. If we had included other multicompartmental parasellar meningiomas that also underwent EED as part of their surgical treatment, the success rates would likely be lower.

It is unlikely that the CNII/CSCN improvements seen in our series were a result of the radiation therapy. None of our patients received radiation prior to surgery. Out of the 12 patients that underwent radiosurgery/radiation therapy, 9 achieved the best outcome in CNII/CSCN function before radiation was started. Even in the three cases where radiation was started before the best CNII/CSCN function was achieved, our end point at 6 months makes it improbable that any improvement of symptoms is a result of the radiation.

All of the three patients in our series who experienced tumor growth on late follow-up did not receive radiation right after surgical treatment was performed.

### Role of the EED

The EED of the CS and/or OC for the management of CS meningiomas seems to have an important role on the preservation or improvement of CNII/CSCN function, especially in an acute and subacute period of symptoms onset. In addition, surgery provides pathological diagnosis in the majority of cases that may affect the radiation treatment plan and prescribed dosing (4).

The analysis of the “EED only” subgroup reinforces the effects of the medial bone decompression on the CSCN and CNII outcomes. The good outcomes with CSCN function in CS meningiomas led us to perform the EED for various types of symptomatic multicompartmental parasellar meningiomas. In this context, the EED is done as part of the endoscopic endonasal stage of tumor resection.

### Study Limitations

Our study has a relatively small number of cases. It is one of the challenges of generating higher quality of evidence when treating rare skull base pathologies. Cavernous sinus meningioma is a rare lesion subjected to treatment selection bias depending on the surgeons training and experience with specific surgical approach or treatment modality. As a consequence, the literature is comprised of similar small series, and it is unlikely that a randomized trial will ever be feasible.

The follow-up length may also be perceived as a relative limitation when assessing therapeutic results of slow growing benign tumors. However, we believe that this is not a major limitation considering that the main goal of our study was to evaluate the role of a conservative surgical decompression on preserving/improving cranial nerve deficit while radiation controls tumor growth.

## Conclusion

The EED for CS meningiomas is a valuable technique when addressing acute/subacute CNII and CSCN deficits. This conservative surgical approach showed good functional outcomes, low morbidity, and low complication rates. However, surgical decompression of the CS does not exempt the need for radiosurgery/radiation therapy for tumor growth control. As this strategy valorizes the improvement of neurological function before radiation treatment, we refer to it as “functional skull base surgery.”

## Data Availability Statement

The raw data supporting the conclusions of this article will be made available by the authors, without undue reservation, to any qualified researcher.

## Ethics Statement

The studies involving human participants were reviewed and approved by The Ohio State University Wexner Medical Center - IRB Committee. Written informed consent for participation was not required for this study in accordance with the national legislation and the institutional requirements.

## Author Contributions

AB-F, AS, and DP conceived an designed the analysis. AB-F, BP, AJ, AS, LP, and LD collected the data. AB-F, BP, LP, BO, RC, and DP contributed data or analysis. AB-F, LP, and DP performed the analysis. AB-F and DP wrote the manuscript. All authors contributed to the article and approved the submitted version.

## Conflict of Interest

RC is a paid consultant for Medtronic. DP is a paid consultant for Medtronic and Stryker and receives Royalties from KLS-Martin. The remaining authors declare that the research was conducted in the absence of any commercial or financial relationships that could be construed as a potential conflict of interest.

## References

[1] DeMonte F Smith HK Al-Mefty O. Outcome of aggressive removal of cavernous sinus meningiomas. J Neurosurg. (1994) 81:245–51. 10.3171/jns.1994.81.2.0245 8027808

[2] O'Sullivan MG van Loveren HR Tew JM. The surgical resectability of meningiomas of the cavernous sinus. Neurosurgery. (1997) 40:238–44. 10.1097/00006123-199702000-00003 9007855

[3] Correa SFM Marta GN Teixeira MJ. Neurosymptomatic carvenous sinus meningioma: a 15-years experience with fractionated stereotactic radiotherapy and radiosurgery. Radiat Oncol. (2014) 9:27. 10.1186/1748-717X-9-27 24438670 PMC3904186

[4] Park K-J Kano H Iyer A Liu X Tonetti DA Lehocky C. Gamma knife stereotactic radiosurgery for cavernous sinus meningioma: long-term follow-up in 200 patients. J Neurosurg. (2018) 1:1–10. 10.3171/2018.2.JNS172361 30028261

[5] Klinger DR Flores BC Lewis JJ Barnett SL. The treatment of cavernous sinus meningiomas: evolution of a modern approach. Neurosurg Focus. (2013) 35:E8. 10.3171/2013.9.FOCUS13345 24289133

[6] Larson JJ van Loveren HR Balko MG Tew JM. Evidence of meningioma infiltration into cranial nerves: clinical implications for cavernous sinus meningiomas. J Neurosurg. (1995) 83:596–9. 10.3171/jns.1995.83.4.0596 7545742

[7] Nanda A Thakur JD Sonig A Missios S. Microsurgical resectability, outcomes, and tumor control in meningiomas occupying the cavernous sinus. J Neurosurg. (2016) 125:378–92. 10.3171/2015.3.JNS142494 26745483

[8] Akutsu H Kreutzer J Fahlbusch R Buchfelder M. Transsphenoidal decompression of the sellar floor for cavernous sinus meningiomas: experience with 21 patients. Neurosurgery. (2009) 65:54–62. 10.1227/01.NEU.0000348016.69726.A6 19574825

[9] Couldwell WT Kan P Liu JK Apfelbaum RI. Decompression of cavernous sinus meningioma for preservation and improvement of cranial nerve function. Technical note. J Neurosurg. (2006) 105:148–52. 10.3171/jns.2006.105.1.148 16871891

[10] Lobo B Zhang X Barkhoudarian G Griffiths CF Kelly DF. Endonasal endoscopic management of parasellar and cavernous sinus meningiomas. Neurosurg Clin N Am. (2015) 26:389–401. 10.1016/j.nec.2015.03.004 26141358

[11] Sivakumar W Barkhoudarian G Lobo BM Zhang X Zhao F Eisenberg A. Strategy and technique of endonasal endoscopic bony decompression and selective tumor removal in symptomatic skull base meningiomas of the cavernous sinus and meckel's cave. World Neurosurg. (2019) 131:e12–22. 10.1016/j.wneu.2019.06.073 31226453

[12] Beer-Furlan A Evins AI Rigante L Anichini G Stieg PE Bernardo A. Dual-Port 2D and 3D endoscopy: expanding the limits of the endonasal approaches to midline skull base lesions with lateral extension. J Neurol Surg B Skull Base. (2014) 75:187–97. 10.1055/s-0033-1364165 25072012 PMC4078187

[13] de Lara D Ditzel Filho LFS Prevedello DM Carrau RL Kasemsiri P Otto BA. Endonasal endoscopic approaches to the paramedian skull base. World Neurosurg. (2014) 82:S121–9. 10.1016/j.wneu.2014.07.036 25496623

[14] Ferrareze Nunes C Beer-Furlan A Doglietto F Carrau RL Prevedello DM-S. The McConnell's capsular arteries and their relevance in endoscopic endonasal approach to the sellar region. Oper Neurosurg. (2018) 14:171–7. 10.1093/ons/opx107 29040718

[15] Kassam AB Prevedello DM Carrau RL Snyderman CH Gardner P Osawa S. The front door to meckel's cave: an anteromedial corridor via expanded endoscopic endonasal approach- technical considerations and clinical series. Neurosurgery. (2009) 64:71–82. 10.1227/01.NEU.0000335162.36862.54 19240575

[16] Labib MA Prevedello DM Fernandez-Miranda JC Sivakanthan S Benet A Morera V. The medial opticocarotid recess: an anatomic study of an endoscopic “key landmark” for the ventral cranial base. Neurosurgery. (2013) 72:66–76. 10.1227/NEU.0b013e318271f614 23271222

[17] Griffiths CF Cutler AR Duong HT Bardo G Karimi K Barkhoudarian G. Avoidance of postoperative epistaxis and anosmia in endonasal endoscopic skull base surgery: a technical note. Acta Neurochir. (2014) 156:1393–401. 10.1007/s00701-014-2107-8 24809531

[18] Radhakrishnan K Mokri B Parisi JE O'Fallon WM Sunku J Kurland LT. The trends in incidence of primary brain tumors in the population of rochester, minnesota. Ann Neurol. (1995) 37:67–73. 10.1002/ana.410370113 7818260

